# A controlled study of the hygienic technical evaluation of the transaxillary approach for inflation-free single-port lumpectomy versus conventional transcervical anterior open surgery in radical thyroid cancer resection

**DOI:** 10.1186/s12957-024-03445-y

**Published:** 2024-06-27

**Authors:** Jie Chen, Bo Xu BM, Chaojie Zhang BM, Chengquan Ma, Tianwen Lu

**Affiliations:** Thyroid and Breast Surgery, Department of General Surgery, Wanbei Coal and Electricity Group General Hospital, Su Zhou, Anhui Province China

**Keywords:** Papillary thyroid cancer, Transaxillary approach, Lumpectomy, Economic characteristics, Social adaptability

## Abstract

**Objective:**

To evaluate sanitary techniques for radical thyroid cancer surgery via the transaxillary approach without inflation single-port endoscopic surgery (TAWISES) and the conventional open anterior cervical approach (COACAS) in a controlled manner.

**Methods:**

This work was a retrospective analysis of the clinical data of 60 patients admitted to our hospital for unilateral radical thyroid cancer surgery between 01/2021 and 12/2022. The control group underwent COACAS (30 patients), and the experimental group underwent TAWISES (30 patients). The patients’ operative time, intraoperative bleeding volume, 24-h postoperative pain index, drainage tube carrying time, hospitalization duration and complication rate were compared and analyzed. The patients were followed up for 3, 6 and 12 months postoperatively and evaluated based on numbness, muscular tightness, pain and other discomfort in the neck, as well as satisfaction with social adaptation and cosmetic incisions. The recurrence status was assessed for 1 year in both groups of patients. A questionnaire survey was conducted to assess patient acceptance of the two surgical approaches. The economic characteristics (cost-effectiveness and cost-utility) of the different approaches in our region were evaluated comprehensively.

**Results:**

The length of the incision, drainage tube carrying time and hospitalization duration were greater in the experimental group than in the control group (*P* < 0.05). The differences in complication rate, intraoperative bleeding volume, 24-h postoperative pain index and recurrence rate were not statistically significant between the two groups (*P* > 0.05). Neck discomfort was greater in the control group, and the difference was statistically significant at the 3-month postoperative follow-up (*P* < 0.05). The differences at the 6- and 12-month postoperative follow-ups were not statistically significant (*P* > 0.05). However, mild discomfort was significantly more common in the experimental group (63.33% > 36.67%, 80% > 53.33%, *P* < 0.05). The experimental group had better social adaptability, greater total medical costs, and better overall patient medical satisfaction than did the control group (*P* < 0.05). The acceptance of TAWISL was greater than that of COACAS (*P* < 0.05).

**Conclusion:**

Compared with COACLAS, TAWISES is safe and effective and better meets the cosmetic, psychological and social adaptation needs of patients. TAWISES is also more cost effective and can be better utilized for the population in our region, filling the gap in surgical modalities for thyroid cancer in in our region.

**Supplementary Information:**

The online version contains supplementary material available at 10.1186/s12957-024-03445-y.

## Introduction

The complex living environment, increased social stress and changes in people’s dietary structure brought about by economic development have contributed to the increasing incidence of various diseases. The innovation of medical technology and increasing health awareness among the population have contributed to increases in the detection rate of diseases. According to the data provided by GLOBOCAN, 586,202 cases of thyroid cancer (TC) were registered in 2020 [1, 2]. The incidence and mortality of TC are expected to increase by 29.9% and 67%, respectively, by the year 2040 [2].

As the representative malignancy with the fastest increasing incidence and detection rate, Papillary thyroid cancer(PTC), as the most common TC, is characterized by a young age, predominantly female patients, and a good prognosis [3]. Although traditional open surgery remains the “gold standard” of treatment [4], residual neck scars are a consistent source of patient dissatisfaction.

Since the first report of lumpectomy thyroid surgery in 1997, various endoscopic thyroid surgery approaches have emerged to reduce the physical and psychological impact of scarring on patients [5]. Based on continuous proficiency in surgical operations, the indications for lumpectomy thyroid surgery have expanded from benign tumors to PTC, and the types of access have expanded to include full areolar access, transoral vestibule access, and transaxillary access. However, the safety and effectiveness of lumpectomy for treating malignant tumors remain controversial and need to be further confirmed [6–8].

Among the many available endoscopic thyroid surgery approaches, the transaxillary approach without inflatable single-port endoscopic surgery (TAWISES) for radical thyroid cancer involves scarring of the axilla. The incision is concealed to meet the cosmetic needs of patients. Therefore, it is the most widely used and accepted technique by surgeons [9]. However, the thoroughness of TC lymph node dissection, overall postoperative patient sensory satisfaction and comfort, and even health economic characteristics are subject to debate. In this study, we focused on the health technology relevance of lumpectomy for radical thyroid cancer via an axillary single-port approach without inflation compared to that of conventional open anterior cervical approach surgery(COACAS) for radical thyroid cancer in our region.

## Materials and methods

### Patients and inclusion criteria

We retrospectively analyzed the clinical data of 99 patients who underwent surgical treatment for unilateral papillary thyroid cancer admitted to our hospital between January 2021 and December 2022. This project was approved by the ethics committee of Wanbei Coal and Electricity Group General Hospital (No. WBZY-LLWYH-2024-019). We certify that the study was performed in accordance with the 1964 declaration of HELSINKI and later amendments. All procedures in this study were conducted in accordance with the Ethics Committee of Wanbei Coal and Electricity Group General Hospital. All patients were informed of the purpose, modality, precautions, and possible complications before enrollment and written informed consent was obtained from all the participants prior to the enrollment of this study.

### Inclusion criteria

①All patients had a unilateral solitary thyroid mass, were initially considered to have thyroid cancer by preoperative ultrasound or fine needle aspiration biopsy (FNAB) and were confirmed to have PTC by routine postoperative pathology.

②Thyroid lesions with a tumor diameter ≤ 2 cm.

③No invasion of the thyroid peritoneum, trachea, esophagus, superior laryngeal nerve or laryngeal recurrent nerve (the patient had no hoarseness and normal vocal cord movement on laryngoscopy, no choking or coughing from drinking and no weakness of pronunciation). Moreover, the patients were free of lateral neck or distant metastasis.

④The patient had high cosmetic requirements, requested relevant surgical treatment after doctor‒patient communication and signed the informed consent form.

### Exclusion criteria

①Patients who were in poor general condition or had other underlying conditions and who could not tolerate surgical procedures.

②Patients with a history of previous neck and affected chest wall surgery or radiotherapy.

③ Patients who were considered to have bilateral or unilateral thyroid cancer with lateral neck lymph node metastasis.

④Tumors larger than 2 cm or tumors with invasion into other organs immediately above the upper pole into the larynx.

If the patient refused treatment, was lost to follow-up or had other diseases, the patient was automatically excluded from the group. Finally, the patients in the control group were subjected to COACAS. A total of 36 patients were enrolled. Patients in the experimental group underwent TAWISES. A total of 36 patients were enrolled.

The general and clinical data (operative time, hospitalization time, 24-h postoperative pain index, tumor size, clinical cost, etc.) of the two groups were compared and analyzed; postoperative satisfaction; discomfort, such as neck pain and numbness; postoperative disease status; and the impact of scar contracture on daily life were investigated. All patients were followed up for 1 year.

We investigated the acceptability of the surgery in terms of cosmetic results, surgical safety, neck discomfort, total hospitalization costs, and acceptance of TAWISES versus COACAS in our region by analyzing 300 patients who visited our department for thyroid disease.

### Surgical procedures

To reduce bias, the surgeries on patients in both the experimental and control groups were performed by the same group of (primary and one assistant) surgeons. The data were collected, evaluated, and registered by the same 2 persons during and after the operation. In case of differences, the senior physician evaluated and confirmed the data.

#### Experimental group

**Fig. 1:**
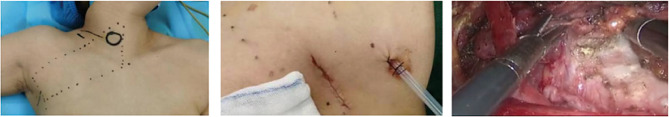
Transaxillary approach inflation-free single-port lumpectomy for radical thyroid cancer

After successful general anesthesia, the patient was placed in a supine position with a pillow under the shoulder, and the head was slightly tilted to the healthy side. The upper arm on the affected side was fully abducted to expose the axilla. An approximately 3–5 cm long incision was made in the skin (the window of endoscope and forceps), and the skin was separated along the surface of the pectoral muscle under direct vision. Then, a 5 mm incision was made ventral to the incision about the anterior axillary line to implant a 5 mm trocar (an ultrasonic knife implantation window). The sternocleidomastoid muscle was exposed with the aid of a retractor. The cavity was entered through the gap between the sternal and clavicular heads of the sternocleidomastoid muscle. The scaphoid hyoid muscle was explored and further separated. The anterior cervical strap muscle was pulled up with a noninflated transaxillary retractor. The thyroid gland was fully exposed, and the true and false envelopes were separated. The recurrent laryngeal nerve was found and exposed. Then, the superior-middle-inferior pole of the thyroid gland was disconnected by ultrasonic knife electrocoagulation. The superior parathyroid gland and blood supply were preserved in situ as much as possible. The lymph nodes in the VI area were cleared along the recurrent laryngeal nerve after routine intraoperative freezing examination revealed PTC. We focused on preserving the inferior parathyroid gland and its blood supply. The trauma surface was definitively hemostatic, and the surgical cavity was rinsed with sterile water. The instruments were inventoried, and a drainage tube was placed after the operation. (As shown in Fig. 1)

#### Control group

**Fig. 2:**
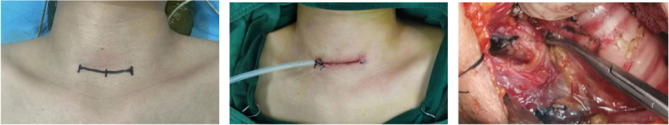
Traditional open surgery through the anterior neck for radical thyroid cancer

After satisfactory anesthesia, the patient was placed in a supine position with the back of the shoulder padded and the head slightly tilted back to expose the neck. A transverse incision approximately 5 cm in length was made on the sternum. The skin and the subcutaneous and broad neck muscles were cut, and the flap was dissociated. The midline was incised, and the affected anterior cervical strap muscle was freed to reveal the affected thyroid gland. The affected laryngeal nerve was free and exposed. The upper, middle and upper pole vessels of the affected thyroid gland were dissociated and ligated, and the affected thyroid gland was excised. The lymph nodes in the VI area were removed for routine pathology after routine freezing confirmed PTC. After the operation, a pressure drainage tube was placed in the thyroid fossa on the affected side (as shown in Fig. 2).

### Observation indicators

The following parameters were observed: (1) the operative time, which was defined as the time from skin incision to suture completion; (2) intraoperative bleeding; (3) postoperative complications (postoperative bleeding, infection, nerve injury, etc.) and the postoperative pain index at 24 h (VAS score: 0, no pain; 1–3, mild pain; 4–6, moderate pain; and 7–10, severe pain); (4) drainage tube carrying time (experimental group drainage fluid ≤ 20 ml, control group drainage fluid ≤ 10 ml, drainage tube was removed); (5) total hospitalization and total cost to patients; (6) neck discomfort for 3, 6 and 12 months after surgery; (7) recurrence and metastasis status of patients in both groups after one year; and (8) patient satisfaction and the impact of the two surgical procedures on patients’ daily social life. Satisfaction was rated based on the Numerical Rating Scale (NRS). NRS was used to evaluate the patients’ satisfaction level. The satisfaction level was rated from 1 to 7. A higher score indicated higher patient satisfaction. The efficacy and treatment cost acceptability of radical thyroidectomy were assessed.

### Statistical methods

SPSS 26 statistical software was used for data analysis. The measurement data are expressed as the mean ± standard deviation (x ± s), and a t test was used to compare normally distributed data; a nonparametric test was used to compare nonnormally distributed data, and the X^2^ test was used to compare discrete data, such as the number of patients (n) and rate (%); *P* < 0.05 indicated a statistically significant difference. Missing values were processed using mean padding; outliers were detected using standard deviation methods for identification and processing.

## Results

**Fig. 3:**
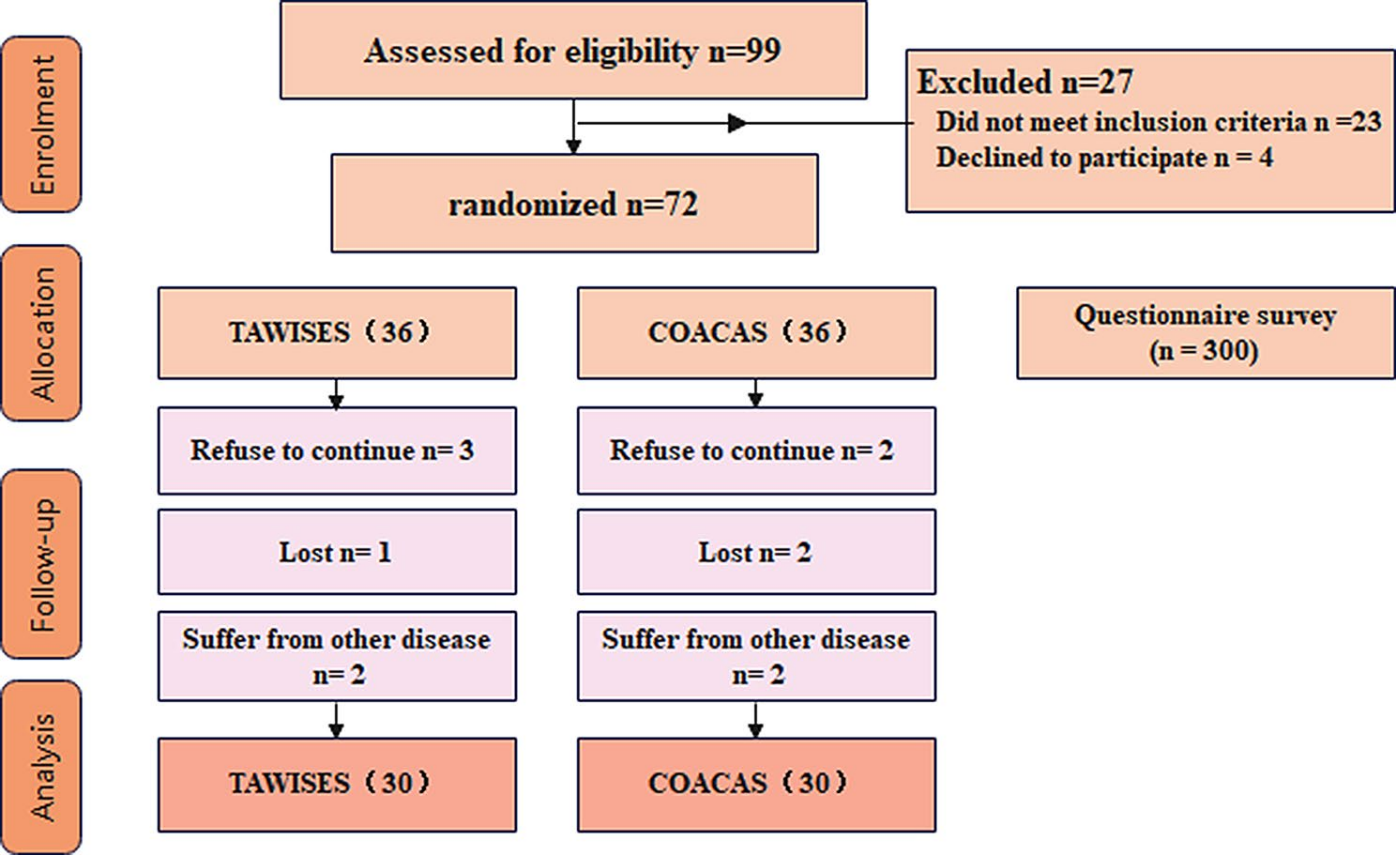
Flow diagram

3.1 A total of 36 patients underwent COACAS. Two patients refused to participate, 2 patients were lost to follow-up, and 2 patients were suffering from other diseases. Finally, 6 patients were excluded, and 30 patients were eligible for enrollment. A total of 36 patients underwent TAWISES. Three patients refused to participate, 1 patient was lost to follow-up, and 2 patients were suffering from other diseases. Finally, 6 patients were excluded, and 30 patients were eligible for enrollment. (As shown in Fig. 3)

The general data (gender, age, and tumor size) of the two groups were not significantly different (*P* > 0.05) and were comparable (as shown in Table 1).

**Table 1:** Comparison of general data between the two groups

Basic information	Experimental group	Control group	X^2/t^	*P* Value
Male/female	1(3.33)	3(10.00)	0.27	0.61
Age(year)	36.3 ± 9.62	41.27 ± 10.45	-1.91	0.6
Tumor diameter(mm)	6.27 ± 3.16	6.60 ± 3.59	-0.38	0.7

The surgery was completed successfully in both groups. The operative time, drainage tube carrying time, hospitalization, postoperative satisfaction and total hospital cost were significantly greater in the experimental group than in the control group. The difference was statistically significant (*P* < 0.05). The difference was not statistically significant (*P* > 0.05) for intraoperative bleeding volume or the pain index 24 h postoperatively (as shown in Table 2).

**Table 2:** Comparison of general data between the two groups

Clinical data	Experimental group	Control group	X^2/Z^	*P* value
Intraoperative bleeding(ml)	13.5 ± 3.97	12.73 ± 3.83	0.67	0.45
drainage tube carrying time(days)	6.43 ± 1.74	4.70 ± 0.87	4.75	< 0.001
hospitalization(days)	11.00 ± 2.75	9.07 ± 1.57	3.34	0.002
Operative Time(min)	149.67 ± 36.22	94.20 ± 24.53	-4.66	< 0.001
VAS score after operation(24 h)	3.73 ± 1.34	3.97 ± 1.77	0.58	0.57
Satisfaction	5.47 ± 1.20	4.73 ± 1.02	2.56	0.013
Total cost of hospitalization(rmb)	18177.90 ± 1566.81	14902.37 ± 990.51	9.68	< 0.001

Comparison of complications between the two groups revealed 1 patient (3.33%) with incisional infection and 2 patients (6.67%) with visible parathyroid tissue without paresthesia or convulsions on postoperative pathology in the experimental group. One patient (3.33%) with incisional infection and 1 patient (3.33%) with postoperative bleeding from the tumor bed were included in the control group. Symptoms of supraglottis, laryngeal regurgitation, or tracheoesophageal injury were absent in either group. One year of follow-up revealed no cases of metastasis or recurrence in either the experimental or control groups. Complications, recurrence or metastasis rates did not significantly differ between groups (*P* > 0.05, as shown in Table 3).

**Table 3:** Comparison of complications and metastatic recurrence rates between the two groups(%)

Complications / Recurrent metastasis		Experimental group	Control group	X^2^	*P* value
Complications	Bleeding	0	1(3.33)	0.22	0.64
	infection	1(3.33)	1(3.33)		
	Injury of parathyroid gland	2(6.67)	0		
	Injury of superior laryngeal nerve	0	0		
	Injury of recurrent laryngeal nerve	0	0		
Recurrent metastasis		0	0		

The postoperative follow-up of patients mainly included subjective feelings (neck discomfort, including skin numbness, muscle tightness, pain and other discomfort) and the impact on objective social life functions.

### Subjective feelings

The follow-up revealed that patients in both groups had different degrees of neck discomfort (including skin numbness, muscle tightness, pain, etc.) at 3, 6 and 12 months after surgery. The degree of discomfort in the experimental group was significantly lower than that in the control group at 3 months after surgery (*P* < 0.05). The difference at 6 and 12 months was not statistically significant (*P* > 0.05), but the proportion of mild discomfort in the experimental group was significantly greater than that in the control group (63.33% > 36.67%, 80.00% > 53.33%), and the difference was statistically significant (*P* < 0.05, as shown in Table 4).

**Table 4:** Qualitative comparison of neck discomfort in the experimental group and control group

Follow-up time	Subjective feelings	Experimental group	Control group	X^2/Z^	*P* value
3 month	Mild	20	10	7.26	0.023
	Moderate	6	15		
	Heavy	4	5		
6 month	Mild	19	11	5.02	0.097
	Moderate	7	15		
	Heavy	4	4		
12 month	Mild	24	16	5.21	0.074
	Moderate	4	11		
	Heavy	2	3		
6 month	Mild	19	11	4.27	0.039
	Moderate /Heavy	11	19		
12 month	Mild	24	16	4.8	0.028
	Moderate /Heavy	6	14		

### Impact on objective social life functions

The impact of complications or scars caused by different surgical procedures for this disease on patients’ daily social life status was analyzed. According to the severity, 4 grades were classified: no effect, mild effect, effect, and severe effect. The experimental group had better social adaptability, and the difference was statistically significant (*P* < 0.05, as shown in Table 5).

**Table 5:** Comparison of the effect on daily social life between the two groups

Follow-up time	Objective social life functions	Experimental group	Control group	X^2/Z^	*P* value
1 year	No effect	13	0	25.33	< 0.001
	Mild effect	15	15		
	effect	2	14		
	Heavy effect	0	1		

To assess the acceptability of TAWISES, 300 patients who visited our department to be treated for thyroid cancer completed a questionnaire survey. The results of this survey indicated that patients were satisfied with the safety, cosmetic outcomes and cost of TAWISES, and the procedure was considered cost effective (Table 6).

**Table 6:** Outpatient flow survey of the acceptance of the transaxillary approach without inflation single-port lumpectomy for thyroid cancer radical surgery

safety	Agreed	Not sure	Disagreed
Number Proportion(%)	209	58	33
	69.67	19.33	11.00
**cost**	**Acceptable**	**Unacceptable**	
Number	258	42	
Proportion(%)	86	16	
**Beauty**	**Satisfied**	**General**	**Not satisfied**
Number Proportion(%)	257	28	15
	85.67	9.33	5.00

## Discussion

Thyroid cancer is the most common malignancy of the head and neck, and its incidence is growing rapidly worldwide [6, 10]. In China, the incidence of thyroid malignancies is continuously increasing at a rate of 20% each year. As the most common differentiated thyroid cancer, PTC has a good prognosis, and the key to successful treatment is surgery [10].

Surgery is the “gold standard” of treatment, but it inevitably causes damage to human epidermal tissues. Traditional thyroid cancer surgery involves an exposed incision at the front of the neck, which seriously affects aesthetics and even causes damage to local functions and subjective feelings due to the formation of keloids. The dissection and resuturing of the anterior neck muscles also contribute to increased neck discomfort. Thus, the repair or minimization of skin damage to achieve cosmetically favorable results and further improve patients’ quality of life in a safe and therapeutic manner has remained a surgical research hotspot.

With the increasing popularity of minimally invasive concepts and lumpectomy techniques, lumpectomy techniques have emerged as alternatives to traditional open surgical options. Miccoli et al. performed the first lumpectomy in 1999, and Kang et al. successfully performed the da Vinci lumpectomy in 2009. Thyroid surgeons have explored and expanded the no-inflation/inflation total lumpectomy approach through axillary, transoral and transthoracic approaches in the hope of achieving radical treatment while shifting the incision to a concealed location [11, 12].

The inflatable transaxillary approach can be used to effectively conceal the exposed incision in the axilla while providing radical treatment. However, this approach increases the incidence of complications, such as hypercapnia, supraventricular tachycardia, subcutaneous emphysema, and longer cavity construction and operation times, compared to the noninflatable axillary approach [13]. The transmammary approach involves a longer subcutaneous tunnel, and more flaps are needed for separation. The clavicle and sternal stalk are obscured, making the removal of the posterior thyroid and clearing of lymph nodes in the VI region more difficult [1]. Transoral endoscopic thyroidectomy (TOET) allows bilateral thyroid lesions to be removed, and the parathyroid glands can usually be clearly identified and preserved in situ after elevating the thyroid gland. The lymph nodes in the central region can be cleared by tunneling the laryngeal nerve from top to bottom [14]. However, the use of CO2 insufflation also increases the risk of air thrombosis and hypercapnia and artificially upgrades the incision level (class I to class II), increasing the risk of incision and anterior cervical skin infection [15–17]. At the same time, TOET requires experienced surgeons and assistants. The da Vinci robot can provide a more comfortable surgical environment for the surgeon. It can protect the parathyroid glands and the recurrent laryngeal nerve through three-dimensional view and multiangle movement. However, its learning costs and treatment costs are high, and it has been difficult to popularize in China [18].

Compared with traditional open surgery and other endoscopic surgeries performed to access the thyroid, TAWISES can be used to treat unilateral thyroid lesions with an invisible incision for cosmetic purposes and a high-magnification visualizer to enlarge the surgical field. Moreover, the route of the recurrent laryngeal nerve and the operator’s view are essentially parallel. The surgeon only needs to surgically dissociate the peritoneum of the thyroid gland to identify the recurrent laryngeal nerve well. This procedure is considered safe and effective.

According to the clinical data, the operative time, drainage tube carrying time, and hospitalization duration were prolonged in the experimental group. This finding was attributed to the free flap construction of the cavity through the axilla to the anterior neck. Neck discomfort was lower in the experimental group than in the control group at 3 months postoperatively and was similar at 6 and 12 months. However, the percentage of patients with mild discomfort was lower and the percentage of patients who were satisfied was greater in the experimental group. The reason for this difference may be related to the different surgical access methods used in the two groups. In the experimental group, the thyroid gland was free and removed through the natural anterior cervical space (between the sternocleidomastoid head and the clavicular head) without opening the cervical white line or the broad cervical muscle. This procedure can reduce scar adhesions in this area, preserve some levels of neck mobility and extension, and greatly reduce neck numbness and swallowing linkage discomfort.

Moreover, the small and hidden incision made during the TAWISES procedure can meet the cosmetic needs of patients, and the reduced postoperative psychological burden of patients can help them better adapt to social life (*P* < 0.05). Patient satisfaction was greater in the experimental group, and the complication rate, intraoperative bleeding, 24-h postoperative pain index and recurrence rate were not significantly different between the two groups. The experimental group had better medical satisfaction and social adaptability than the control group. This conclusion is consistent with the results of Wirth U and Lee MC [12, 13]. The total cost of hospitalization increased accordingly because of the use of lumpectomy instruments, special retractor hooks and ultrasonic knives. In our daily medical practice, we often encounter cases in which patients cannot choose the best and most appropriate technical option because of the high cost. The data in this study showed that the cosmetic needs of patients was met in the experimental group, but the overall cost increased significantly in this group compared to that of the control group. Based on 300 patients who visited our department for thyroid disease, the economic cost of TAWISES was as high as 86%, and the acceptability of TAWISES was greater than that of COACAS (*P* < 0.05). Therefore, TAWISES meets economic level requirements of our region.

This study has the drawback of a small sample size, and the data of neither group completely reflected the advantage of transaxillary cavernous thyroid surgery to protect the laryngeal recurrent nerve (*P* > 0.05). In addition, patients did not have symptoms of hypocalcemia because this study involved unilateral thyroidectomy. However, the parallel magnified view made it safer and more convenient to find and protect the parathyroid glands and their blood supply. Such advantages have been reported in related domestic and international studies. The study data showed two cases of postoperative pathology in the experimental group in which some of the parathyroid tissue was visible, both of which occurred when the technique was first introduced to our institution (2020); thus, this finding was attributed to a lack of skill on the part of the surgeon and assistant.

In conclusion, TAWISES is safer and more effective than COACAS. A concealed incision can better meet the cosmetic, psychological and social adaptation needs of patients. Therefore, this technique is more acceptable to patients. In terms of economics, the cost of lumpectomy is within the range of acceptance and has more satisfactory utility for the population in our region, filling the gap in surgical modalities for thyroid cancer in our region.

### Electronic supplementary material

Below is the link to the electronic supplementary material.

Supplementary Material 1

Supplementary Material 2

Supplementary Material 3

Supplementary Material 5

## Data Availability

The datasets used or analyzed during the current study are available from the corresponding author on reasonable request.
